# Individual Differences in Children’s (Language) Learning Skills Moderate Effects of Robot-Assisted Second Language Learning

**DOI:** 10.3389/frobt.2021.676248

**Published:** 2021-08-24

**Authors:** Rianne van den Berghe, Ora Oudgenoeg-Paz, Josje Verhagen, Susanne Brouwer, Mirjam de Haas, Jan de Wit, Bram Willemsen, Paul Vogt, Emiel Krahmer, Paul Leseman

**Affiliations:** ^1^ Department of Development of Youth and Education in Diverse Societies, Utrecht University, Utrecht, Netherlands; ^2^ Section Leadership in Education and Development, Windesheim University of Applied Sciences, Almere, Netherlands; ^3^ Amsterdam Center for Language and Communication, University of Amsterdam, Amsterdam, Netherlands; ^4^ Department of Modern Languages and Cultures, Radboud University, Nijmegen, Netherlands; ^5^ Department of Cognitive Science and Artificial Intelligence, Tilburg University, Tilburg, Netherlands; ^6^ Department of Communication and Cognition, Tilburg University, Tilburg, Netherlands; ^7^ Department of Intelligent Systems, KTH Royal Institute of Technology, Stockholm, Sweden; ^8^ School of Communication, Media and IT, Hanze University of Applied Sciences, Groningen, Netherlands

**Keywords:** social robots, second language learning, child-robot interaction, individual differences, (language) learning skills

## Abstract

The current study investigated how individual differences among children affect the added value of social robots for teaching second language (L2) vocabulary to young children. Specifically, we investigated the moderating role of three individual child characteristics deemed relevant for language learning: first language (L1) vocabulary knowledge, phonological memory, and selective attention. We expected children low in these abilities to particularly benefit from being assisted by a robot in a vocabulary training. An L2 English vocabulary training intervention consisting of seven sessions was administered to 193 monolingual Dutch five-year-old children over a three- to four-week period. Children were randomly assigned to one of three experimental conditions: 1) a tablet only, 2) a tablet and a robot that used deictic (pointing) gestures (the no-iconic-gestures condition), or 3) a tablet and a robot that used both deictic and iconic gestures (i.e., gestures depicting the target word; the iconic-gestures condition). There also was a control condition in which children did not receive a vocabulary training, but played dancing games with the robot. L2 word knowledge was measured directly after the training and two to four weeks later. In these post-tests, children in the experimental conditions outperformed children in the control condition on word knowledge, but there were no differences between the three experimental conditions. Several moderation effects were found. The robot’s presence particularly benefited children with larger L1 vocabularies or poorer phonological memory, while children with smaller L1 vocabularies or better phonological memory performed better in the tablet-only condition. Children with larger L1 vocabularies and better phonological memory performed better in the no-iconic-gestures condition than in the iconic-gestures condition, while children with better selective attention performed better in the iconic-gestures condition than the no-iconic-gestures condition. Together, the results showed that the effects of the robot and its gestures differ across children, which should be taken into account when designing and evaluating robot-assisted L2 teaching interventions.

## Introduction

The current study addresses the use of social robots in language education. Specifically, we investigated how individual differences among children affect the added value of social robots for teaching second language (L2) vocabulary to young children. While studying the effects of robots is in itself important in view of applications in education, it is crucial to compare the effectiveness of robots to that of cheaper and more accessible technological aids such as tablets. Several potential advantages of robots relative to other technologies such as tablets have been identified in extant research. For example, social robots allow for interactions that make use of the physical environment (e.g., acting upon objects, enacting particular movements or operations, using various types of gestures) and they can stimulate more natural, human-like interactions because of their humanoid appearance (Belpaeme et al., 2018; van den Berghe et al., 2019). The use of iconic gestures is known to support L2 vocabulary learning (Tellier, 2008; Macedonia et al., 2011; Rowe et al., 2013), and a robot’s iconic gestures and other non-verbal cues have been found to benefit learners as well (Kory Westlund et al., 2017; de Wit et al., 2018).

Current evidence on the effectiveness of robot-assisted language learning (RALL), however, is mixed (see for reviews Kanero et al., 2018; van den Berghe et al., 2019), and there is inconclusive evidence on the possible benefits of robots over other forms of technology (Han et al., 2008; Hyun et al., 2008; Leyzberg et al., 2012; Gordon et al., 2015; Kory Westlund et al., 2015; Zhexenova et al., 2020). Specific for word learning, positive effects of robots on learning were found in several single-session studies (Tanaka and Matsuzoe, 2012; de Wit et al., 2018), while only moderate learning gains were found in multiple-session studies (Kanda et al., 2004; Gordon et al., 2016). This effect is in contrast with evidence regarding effective and impactful vocabulary training programs involving human tutors where multiple sessions with a large number of repeatedly presented words are usually more effective than single sessions (Marulis and Neuman, 2010). Perhaps this difference is due to the novelty effect: If children have little or no experience with robots, they may attend more to the robot and become more motivated by it, and thus learn more than when they become more familiar with robots (see Leite et al., 2013, for an overview of long-term interactions with robots). Multiple-session studies are thus required to rule out a short-lived novelty effect as a main cause of children’s word learning in RALL studies.

Moreover, and crucial to this paper, there is evidence that RALL may be only effective for a subgroup of children (such as children who are motivated to play with the robot; Kanda et al., 2004), suggesting that individual characteristics of children may moderate the effects of RALL. It is possible that robots are useful language-education tools for certain children only, for example, depending on children’s prior language knowledge and general (language) learning abilities. However, studies on the role of individual child characteristics in RALL, enabling the identification of such specific groups, are scarce. Most studies on adaptive learning focus on learners’ age, gender, or cognitive or affective state during the learning task (e.g., Gordon et al., 2016; Ahmad et al., 2019), and not so much on learners’ prior skills.

The current study, therefore, aims to add to the evidence regarding the effectiveness of robots in L2 teaching of young children in a vocabulary training spanning multiple sessions, by specifically focusing on the role of individual differences across children in skills related to the task at hand. We focused on three skills suggested by the literature to play an important part in language learning: children’s first language (L1) vocabulary knowledge (Wolter, 2006), phonological short-term memory capacity (Gathercole and Baddeley, 1990; Service, 1992; Baddeley et al., 1998; Masoura and Gathercole, 2005; Gathercole, 2006; Verhagen et al., 2019), and selective attention (Schmidt, 1990; Robinson, 1995). We will examine whether these skills moderate any effects of RALL on children’s learning of L2 words.

The current study follows up on a previous study using the same data (Vogt et al., 2019). In this previous study, the added value of a social robot and its iconic gestures for L2 vocabulary learning were investigated. Native Dutch-speaking five-year-old children were taught L2 English vocabulary in the domains of mathematical and spatial language in a series of seven short, individually administered lessons. Children were taught words through language games on a tablet in one of three conditions: 1) by themselves (the tablet-only condition); 2) with a robot that used deictic (pointing) gestures (the no-iconic-gestures condition); or 3) with a robot that used both deictic and iconic gestures (i.e., gestures depicting the target word; the iconic-gestures condition). In addition, a control group of children was included who did not receive the vocabulary training but played dancing games with the robot instead. Children in the experimental conditions were found to outperform children in the control condition on word-knowledge tasks in two post-tests, both directly after the training and two to four weeks later. However, there were no differences in word knowledge between children across the three experimental conditions on either one of these post-tests. Thus, no overall benefit of the robot’s presence or its iconic gestures was found in Vogt et al. (2019).

In the present study, we extend this earlier study by Vogt et al. (2019) by investigating whether any effects of the robot’s presence or its gestures would be moderated by children’s (language) learning skills. Both the general research question on the added value of the robot and its iconic gestures (answered in Vogt et al., 2019) and the follow-up exploratory question on individual differences (answered in the current paper) were preregistered on AsPredicted^1^. As noted above, we considered three aspects of children’s (language) learning skills, as moderator variables: L1 vocabulary knowledge, phonological memory, and selective attention. If effects of these variables are found, our findings will show the importance of taking into account individual differences in RALL and help tailor RALL to individual children to optimize learning outcomes. Below, we first describe how L1 word knowledge, phonological memory, and selective attention may play a role in L2 word learning, before we turn to our research question and hypotheses on how they may play a role in RALL in particular.

Learning an L2 is dependent on both the quality and quantity of the L2 input (Hoff, 2013; Unsworth, 2016) and on characteristics of the learner (i.e., the learner’s cognitive and personality resources; Cummins, 1991). Prior L1 knowledge may help in L2 learning, as learners can map new L2 labels onto underlying concepts which they already acquired in their L1, provided that concepts are similar (Wolter, 2006). Besides conceptual similarity, similarity in word form between L1 and L2 can also aid in L2 learning, at least when this similarity also entails similarity in meaning (Brenders et al., 2011; Hemsley et al., 2013; Sheng et al., 2016). On the basis of these findings, children with larger L1 vocabularies are expected to learn more words from L2 vocabulary interventions than children with smaller L1 vocabularies. Children with larger L1 vocabularies can use their richer lexical and conceptual networks to disambiguate new input and to integrate it in existing knowledge. This phenomenon, found in particular for reading instruction but also in vocabulary learning (e.g., Penno et al., 2002), has been referred to as the Matthew effect (Stanovich, 2009).

Another factor relevant for L2 learning is children’s phonological memory, defined as the capability to construct a phonological representation of speech sound sequences and to temporarily hold this representation active in memory for further processing (Gathercole and Baddeley, 1990; for a review on the relationship between phonological memory and word learning, see Gathercole, 2006). Phonological memory has been found to predict both L1 and L2 vocabulary learning (Gathercole and Baddeley, 1990; Service, 1992; Baddeley et al., 1998; Masoura and Gathercole, 2005; Gathercole, 2006; Verhagen et al., 2019). Phonological memory may aid L2 vocabulary learning, either directly or through its effect on L1 vocabulary knowledge, in particular if the learner is a novice and still has limited L2 vocabulary knowledge (Cheung, 1996; Masoura and Gathercole, 2005). Learners with substantial L2 vocabulary knowledge can rely on semantic, conceptual, or phonological similarities between novel words and words they have already learned, while novice learners cannot do this and thus have to rely more on their phonological memory (Masoura and Gathercole, 2005).

Finally, language learning in both L1 and L2 may depend on general learning abilities, in particular selective attention – a skill that has been considered the core of executive functions and working memory by some researchers (Garon et al., 2008; Mulder et al., 2014; Hendry et al., 2016; Cowan, 2017). Selective attention, defined as a domain-general, effortful mechanism of perceptual focusing, helps individuals to filter relevant information from irrelevant information in the encoding stage of linguistic information processing and supports processing in working memory. Language learning is thought to depend in part on automatic implicit processes (e.g., statistical learning), but attention can strengthen implicit learning (e.g., Lewkowicz and Hansen-Tift, 2012; Stevens and Bavelier, 2012), and learning may also depend on explicit processes that require attentional effort, especially in L2 learning at a later stage (e.g., the Noticing Hypothesis; Schmidt, 1990).

In the present study, we investigated whether individual differences in L1 word knowledge, phonological memory, and selective attention moderated the extent to which children benefited from the robot’s presence and its iconic gestures during robot-assisted L2 learning. We used the data from Vogt et al. (2019), from all three experimental conditions (the tablet-only, no-iconic-gestures, and iconic-gestures condition), and the control condition. The choice to include a tablet-only condition was motivated by the fact that we had to work around limitations of the robot with regard to speech and object recognition (Kennedy et al., 2017; Wallbridge et al., 2017), which could only be resolved by including a tablet as an additional device for communication and interaction, as is explained more extensively in Vogt et al. (2019). Based on the findings in language learning research, discussed above, we expected that children with larger L1 vocabulary knowledge, larger phonological memory capacity, and a higher level of selective attention would learn more English words across all experimental conditions (i.e., conditions involving a robot and/or a tablet) than children scoring lower on these skills. We did not expect to see effects of L1 vocabulary knowledge, phonological memory, and selective attention for children in the control condition on their English word knowledge, as these children were not taught any English words. We contrasted the experimental conditions with the control condition to make sure that, if any moderator effects were found in the experimental conditions, they would pertain to the learning process, and not to the test taking.

In the remainder or this section, we will discuss our hypotheses regarding possible moderator effects in the robot-assisted vs. tablet-only conditions, before discussing our hypotheses regarding moderator effects in the iconic-gestures vs. no-iconic-gestures conditions. All our hypotheses are quite general, as our study is, to the best of our knowledge, the first to investigate the moderating effects of children’s (language) learning skills on RALL. Our expectation was that the robot conditions offered children a more naturalistic and supportive language learning setting than the tablet-only condition (i.e., a setting in which the learner interacts with another being and which is grounded in the physical environment; Barsalou, 2008; Ellis, 1999; Gallaway and Richard, 1994; Hockema and Smith, 2009; Iverson, 2010; Wellsby and Pexman, 2014), as the robot had a social presence and provided visual input (i.e., iconic and/or deictic gestures) in addition to the tablet. Our hypothesis, therefore, was that the presence of the robot would particularly benefit children who are poorer at language learning, that is, children with smaller L1 vocabulary knowledge, smaller phonological memory capacity, and a lower level of selective attention. These children in particular would need support in relating the novel (L2) words to their existing (L1) knowledge. Thus, we expected these children to show larger differences in learning outcomes between the robot-assisted conditions and the tablet-only condition compared to children higher in (language) learning abilities.

Our hypothesis with respect to the difference between the two robot-assisted conditions (i.e., the added value of the robot’s iconic gestures) was that the iconic gestures would further add to the naturalistic language learning environment and its visual support, and therefore, would particularly benefit children poorer at language learning. Iconic gestures visualize words and help learners to relate novel words to existing concepts (Tellier, 2008; Macedonia et al., 2011; Rowe et al., 2013), which may benefit children poorer in language learning in particular (van Berkel-van Hoof et al., 2019). Thus, we expected children low in (language) learning abilities to show a larger difference in learning outcomes between the iconic-gestures condition and the no-iconic-gestures condition, compared to children with higher learning abilities.

## Methods

### Participants

One hundred and ninety-three^2^ monolingual Dutch preschoolers (95 girls) with an average age of 68.4 months (range 59–81 months, *SD* = 4.7 months) participated in the study. They were recruited from nine different schools in the Netherlands and were randomly assigned within schools to one of the four conditions, while ensuring a similar gender distribution over conditions. None of the schools taught English to preschool children. Parents indicated in a background questionnaire that most children received limited English input. Most children heard, with a maximum of 2 days per week, some English in the media or when parents used stand-alone words and phrases like “let’s go”. Table 1 displays the background characteristics of the children divided over the four conditions. There were no significant differences between conditions in parental education, age, and gender, all *p*s > 0.303. Eleven additional children started the lessons but did not complete them due to illness, technological problems, or because they did not want to participate anymore (*n* iconic-gesture condition = 6, *n* no-iconic-gesture condition = 3, *n* tablet-only condition = 2). Three additional children were pre-tested but excluded from the experiment because they knew more than half of the target words at the pre-test. Informed consent for all children was obtained from parents/caretakers prior to data collection. The L2TOR project, in which this study was embedded, received ethical approval from Utrecht University’s Ethics Committee under protocol number FETC16-039.

**TABLE 1:** Background characteristics of the children in the four conditions.

	Tablet only	No iconic gestures	Iconic gestures	Control
*N*	53	54	54	32
*n* girls	29	26	22	18
*M* age (*SD*) in months	69.1 (4.4)	68.5 (4.7)	68.4 (4.8)	66.9 (4.7)
Age range in months	61–79	59–79	60–81	59–79
*M* standardized PPVT score	105.1 (12.3)	108.6 (11.7)	107.9 (14.5)	108.9 (14.0)
Parental education				
Academic level	60%	72%	74%	66%
Vocational level	33%	26%	20%	24%
Secondary school	7%	2%	6%	10%

*Note.* Information on parental education of both parents was gathered through a questionnaire with a response rate of 65.8%, thus for 127 out of 193 children (*n* iconic-gestures condition = 40, *n* no-iconic-gestures condition = 32, *n* tablet-only condition = 34, *n* control condition = 21).

### Overview of Experimental Sessions

**FIGURE 1:**
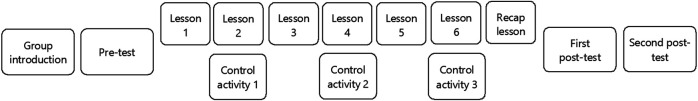
Overview of the experiment.

The experiment consisted of a pre-test, seven tutoring sessions, and two post-tests (see Figure 1 for an overview of the experiment). All children participated in a groupwise introduction prior to the pre-test (see Procedure for more information). In the pre-test, we measured children’s knowledge of the L2 target words and their L1 vocabulary knowledge, phonological memory, and selective attention. The training was administered in one of four conditions, and had a between-subject design. In the experimental conditions, children played language games on a Microsoft Surface tablet: 1) by themselves; 2) with a robot that used deictic gestures (see the Robot section for more information on the robot used); or 3) with a robot that used iconic and deictic gestures. Children received on average two lessons per week over a period of on average 24 days (*SD* = 5.5 days). Children in a fourth, control condition did not play language games but danced with the robot during three sessions, once every week. Children’s immediate learning outcomes were measured in a game concluding each lesson (which are beyond the scope of the current paper), and in a post-test one or two days after the seven tutoring lessons and a second post-test two to four weeks after the first post-test (*M* = 18.9 days, *SD* = 3.6 days) to measure retention over a longer period.

### L2 Vocabulary Lessons

The lesson series consisted of seven individual lessons: six lessons in which new L2 vocabulary was provided, and one recap lesson in which all target words were repeated. Five or six target words were taught within each lesson, resulting in a total of 34 target words. The target words were chosen such that they were part of early mathematical and spatial language. This type of language – academic language – is highly important for later academic success (Hoff, 2013; Leseman et al., 2019). The overall theme of the lesson series was an area to be explored, with different locations for each lesson, such as “the zoo”, “the bakery”, etcetera. The locations were chosen such that they were familiar and relevant to young children. See Table 2 for an overview of the lesson series, the locations, and the target words.

**TABLE 2:** Overview of the lesson series and target words.

Lesson	Location	Target words
One	Zoo	One, two, three, add, more, most
Two	Bakery	Four, five, take away, fewer, fewest
Three	Zoo	Big, small, heavy, light, high, low
Four	Fruit shop	On, above, below, next to, fall
Five	Forest	In front of, behind, walk, run, jump, fly
Six	Playground	Left, right, catch, throw, slide, climb
Seven	Photo book	Repetition of all target words

Each lesson consisted of four parts. First, the child was greeted, a reference was made to the previous lesson, and the location of the current lesson was introduced. Then, the new target words were modelled. New target words were first introduced by a pre-recorded speech sample of a native (Canadian) English speaker. The child was asked to repeat the target word, as this benefits productive recall of target words (Ellis and Beaton, 1993). Then, the child was instructed to perform several tasks on the tablet to practice the target words, for example, during the first lesson in the zoo, children had to put two elephants in a cage to practice the word “two”. The tasks allowing children to practice the target words differed per target word. Some target words required manipulations on the tablet, while others allowed for more physical activity. For example, children were asked to act out running when being taught the word “running”. The lessons concluded with a short test, to measure immediate learning outcomes. We will not discuss these immediate tests in this paper, as this would make our–already extensive–data set too complicated and would distract from the overall picture in which we were interested, namely the overall effect of (language) learning skills on robot-assisted word learning and retention, rather than immediate learning gains.

Each target word was repeated ten times throughout the lesson: nine times by the robot, and once by the native English speaker when it was introduced. Each target word reoccurred once in the following lesson and twice in the recap lesson. During the recap lesson, a photo book appeared on the tablet, which showed print screens from the previous lessons. Children had to practice repeating the target words once more during this recap lesson.

### Robot

The robot used in this study was a Softbank Robotics NAO robot^3^. The robot was sitting in crouch position during the lesson series in a 90° angle to the right of the child, which was sitting on the floor facing the tablet that was positioned on an elevated surface.

**FIGURE 2:**
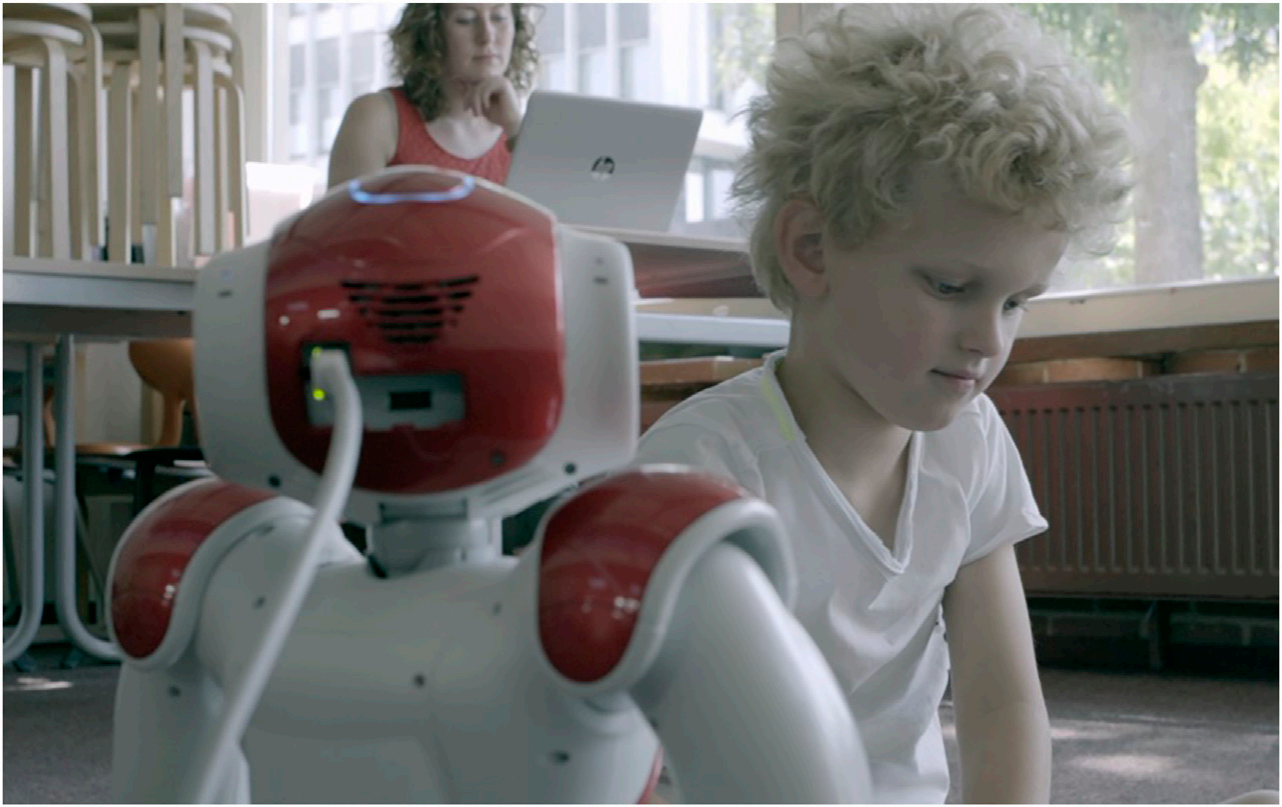
A child engaging in a lesson with the robot.

The robot’s responses had been preprogrammed, such that its responses and behaviors were consistent for all children. The robot was nearly autonomous; it behaved by responding to the child’s actions on the tablet. The only function controlled by the experimenter was voice detection, as automatic speech recognition systems do not work reliably for children (Kennedy et al., 2017). This function was only used when children were asked to repeat the target words. The experimenter indicated, using a graphical user interface on a laptop computer, whether the child had produced sounds or not. The laptop computer was not in direct sight of the child (see Figure 2). The robot was introduced as Robin (which is a gender-neutral name in Dutch), being a peer that was going to learn English words together with the children.

The robot acted as a slightly more knowledgeable peer who understood the game usually faster than the child. As such, the robot performed several behaviors during the training: 1) talking to the child and explaining the tasks of the lesson; 2) pronouncing the target words; 3) providing feedback on the actions of the child; 4) pointing to the tablet while explaining what to do; 5) performing required manipulations in case the child failed to perform a specific task. In case of the latter, the robot moved its arm above the tablet and any required manipulations “magically” occurred. In the iconic-gestures condition, the robot made an iconic gesture each time it pronounced a target word in English. These gestures were modeled after the gestures adults made in a gesture-elicitation procedure when they were asked to make an iconic gesture for each target word^4^.

To ensure that the content and structure of the lessons were the same between the different conditions, in the tablet-only condition, the robot’s voice was redirected through the tablet’s speakers, and the robot itself was hidden from sight. Thus, the robot was used “behind the scenes” to operate the system, but children only saw and interacted with the tablet. In the robot-assisted conditions children thus interacted with the robot and the tablet, whereas in the tablet-only condition they interacted with the tablet only.

### Measures

#### Pre-test Translation Task

To measure whether children knew the L2 English target words prior to the lesson series, we administered a translation task. In this task, children heard the 34 English target words one by one and were asked to translate them to Dutch. The target words were pre-recorded by a native speaker (different from the native speaker whose voice introduced the target words for the first time through the tablet) and played through a laptop computer. Two versions of this task were used, differing in word order. The first list of words was created by listing the target words randomly, and a second list was created by reversing the first list. Children were awarded one point per correct answer, yielding a maximum score of 34 points. Cronbach’s alpha showed that the internal consistency of the task was excellent, α = 0.96.

#### Post-test Translation Tasks

To measure how many L2 English target words the children learned during the lesson series, we administered two translation tasks: one from English to Dutch and one from Dutch to English. The task was the same as the pre-test translation task, except that children now also had to translate the words from Dutch to English. We did not include the Dutch-to-English translation task in the pre-test, because this would make the pre-test too long and difficult for the children (they were expected to know very few English words, and translating them from English to Dutch was expected to be a sufficient measure to assess their existing knowledge of the English target words). Both tasks were administered twice after the lesson series had ended, once during the first post-test and once during the second post-test. Children were awarded one point per correct answer, resulting in a maximum score of 34 points per task. Cronbach’s alpha showed that the consistency of both tasks was excellent, α = 0.94 at the first post-test and α = 0.95 at the second post-test for the English-to-Dutch translation task, and α = 0.97 at the first post-test and α = 0.98 at the second post-test for the Dutch-to-English translation task.

#### Post-test Comprehension Task

We administered a comprehension task to measure children’s receptive knowledge of the target words taught. The comprehension task was a picture-selection task in which we presented children with three images (still photos for most words, or short films in the case of verbs) on a laptop screen. Children then had to select the image corresponding to the target word they heard. Again, pre-recorded speech was used. A bilingual native English-Dutch speaker pronounced a Dutch carrier sentence “waar zie je” (“where do you see”) followed by the target word in English. There were three trials for each target word, with different distractors each time. We selected half of the target words for this task to reduce children’s fatigue, as a comprehension task consisting of all items would have been too long for the children. The target words included in the tests were chosen such that words from each lesson were included and that different types of words (verbs, adjectives, prepositions) were included. Two versions of this task were used, differing in word order: The first list of words was created by listing the target words randomly, and a second list was created by reversing the first list. Children were awarded one point per correct answer, resulting in a maximum score of 54 points. This task was administered during the first and second post-test. Cronbach’s alpha showed that the consistency of the task was good, α = 0.84 at the first post-test and α = 0.87 at the second post-test.

#### L1 Vocabulary

We used the Dutch version of the Peabody Picture Vocabulary Test (PPVT-III-NL, Dunn et al., 2005) to measure children’s Dutch receptive vocabulary knowledge. In this task, children are presented with four pictures and asked to select the picture corresponding to a word said by the experimenter. The task contains a total of seventeen sets, with each set consisting of twelve items. The test is adaptive, such that the starting set is chosen depending on the age of the child, and testing is stopped when the child makes nine or more errors within one set. The test is age-normed, with a mean of 100 and a standard deviation of 15. Cronbach’s alpha is described in the test manual to be between 0.92 and 0.94. We used standardized scores in our analyses.

#### Phonological Memory

The Cross-Linguistic Nonword Repetition Task (CL-NWR) was used to measure phonological memory (Boerma et al., 2015; Chiat, 2015). The CL-NWR is a computerized task appropriate for young children, consisting of sixteen items, ranging from two to five syllables in length. Children hear a previously recorded, non-existing word via a laptop computer, and are asked to repeat it. Children receive two practice items (two one-syllable nonwords) before starting. Children’s responses were scored online by the experimenter and they received one point for each word that they repeated correctly, yielding a maximum score of twelve. Cronbach’s alpha showed that the consistency of this task was satisfactory, α = 0.76. Ten percent of the data was scored independently by an additional researcher based on video recordings of the test. Inter-rater reliability was good with 89% agreement, κ = 0.74 [95% CI (0.663–0.819)], *p* < 0.001.

#### Selective Attention

A computerized visual search task was used to measure selective attention (Mulder et al., 2014). In this task, children were shown a display of animals on a laptop screen consisting of elephants, bears, and donkeys that were similar in color and size. Children were asked to find as many elephants as possible among distractor animals. Children were given three practice items and four test items that increased in difficulty. In the first two test items, 48 animals appeared on a six by eight grid. In the third item, 72 animals (similar in size to the first two test items) appeared on a nine by eight grid. In the last item, 204 animals (smaller in size than in the other three test items) appeared on a 12 by 17 grid. There were eight targets (elephants) in total in each test item. Each test item lasted 40 s. The experimenter encouraged children to search as quickly as possible and gave feedback according to a strict protocol. Elephants that were found were crossed off with a line by the experimenter. The number of targets located correctly per item was calculated and averaged across items, resulting in a maximum score of eight. Cronbach’s alpha showed that the consistency of this task was good, α = 0.86.

### Procedure

#### Group Introduction of Robot

Prior to the individual sessions, the robot was introduced to all children in a group session. The robot introduced itself and did a dance with the children. The groupwise introduction served to familiarize children with the robot, and reduce potential anxiety during the individual sessions.

#### Pre-test

All children were tested individually by a trained experimenter in a quiet room in their schools. Children were administered the tasks in the following order: PPVT, pre-test translation task, selective-attention task, and CL-NWR. Furthermore, a perception questionnaire was administered (also during the first post-test) which measured the degree to which children anthropomorphized the robot. This questionnaire is beyond the scope of the current paper as it did not measure language skills or learning outcomes, and the results of this questionnaire can be found in Van den Berghe et al. (2020). The pre-test session lasted 30–40 min. Children got a sticker in reward for each task.

#### L2 Vocabulary Lessons

Each lesson was administered individually in a quiet room at the children’s schools. At the start of the first session, the experimenter explained how the child could perform the requested actions on the tablet during the lessons (e.g., swiping and tapping), and helped the child to play the game. The experimenter was always present during the lessons to help children if needed, and to control the robot. The lesson could be paused if children needed a break. Each lesson lasted 15–20 min.

#### Control Activities

Children in the control condition participated in a total of three activities with the robot, each administered individually in a quiet room in the children’s schools. In each session, the robot greeted the children, did a dance together with the child, and said goodbye. Each session lasted around five to 10 min.

#### First and Second Post-test

Children were administered the various tasks in the following order: the English-to-Dutch translation task, the Dutch-to-English translation task, and the comprehension task. During the first post-test the anthropomorphism questionnaire was also administered. Each session lasted around 30 min. Children got a sticker in reward for each task completed.

### Analyses

We ran a MANOVA to compare the four groups of children on L1 vocabulary knowledge, phonological memory, selective attention, and pre-test scores. Children’s scores on the comprehension task were compared against chance level (33%) using one-sample *t*-tests. To investigate differences in learning outcomes between the four conditions, we ran mixed-effect logistic regression models in the statistical package R (R Core Team, 2017) using the lme4 package (Bates et al., 2015). Dependent variables were children’s binary (correct/incorrect) scores on the translation tasks and the comprehension task. The analyses were run separately for the translation tasks and the comprehension task, as they were assumed to measure different types of vocabulary knowledge. For both types of tasks, both assessments (the first and second post-test) were included.

Linear mixed-effects models included both fixed and random factors. The fixed-effect factors that were included in the models for the comprehension and translation tasks were condition (control, tablet-only, no-iconic-gestures, and iconic-gestures) and time (first and second post-test), with an interaction between them. For the translation task, target language (from English as source to Dutch as target, and vice versa) was included as an additional fixed-effect factor. The models were run separately for each of the three moderator variables (L1 vocabulary knowledge, phonological memory, and selective attention), as models with more than one moderator variable did not converge. The moderator variables were included as a fixed-effect factor in interaction with condition.

We included random factors and slopes by estimating a series of models with various combinations of random factors and slopes. We compared models by performing likelihood ratio tests that compared the goodness of fit using the ANOVA function in the base package (R Core Team, 2017). First, models were selected by checking whether the *p*-value from the likelihood ratio test was significant. Then, AIC and BIC values were compared, and the model with the smallest values were chosen. For the translation tasks, “participants”, “target words”, and “test item number” were included as random factors, and random slopes for target words (condition*target word). For the comprehension task, “participants”, “target words”, and “test item number” were included as random factors, and no random slopes were included as models including random slopes did not converge. We kept our models maximal, that is, we chose the models with the maximal random effects structure that converged (Barr et al., 2013).

We applied orthogonal sum-to-zero contrast coding to our categorical effects (i.e., condition, time, language; Schad et al., 2020), and all continuous variables (i.e., vocabulary knowledge, phonological memory, selective attention) were centered around zero (Baguley, 2012, pp. 590–621). For time, the first post-test (coded as −0.5) was contrasted with the second post-test (coded as 0.5). For condition, there were three contrasts: Contrast one contrasted the three experimental conditions (each coded as 0.25) with the control condition (coded as −0.75); Contrast two contrasted the two robot-assisted conditions (each coded as −0.33) with the tablet-only condition (coded as 0.66); and Contrast three contrasted the iconic-gestures condition (coded as −0.5) with the no-iconic-gestures condition (coded as 0.5). The number of iterations was increased to 100,000 using the bobyqa optimizer to solve issues of non-convergence (Powell, 2009).

The full results of each model can be found in the Supplementary Tables S2–7)^5^. The “ß” is an indicator of the effect size. To reduce the risk of Type-1 error when conducting multiple comparisons, we applied the Benjamini-Hochberg procedure (Benjamini and Hochberg, 1995) with a false-discovery rate of 5%. The outcomes of this procedure can be found in the Supplementary Tables S8–13), and the calculations can be found online with the dataset^6^.

## Results

### Descriptive Analyses

Table 3 displays the descriptive statistics for all the variables included in the analyses for the children in each condition separately. A MANOVA showed no statistically significant differences in L1 vocabulary, phonological memory, selective attention, and English vocabulary pre-test scores between the conditions, *F* (12, 397) = 1.75, *p* = 0.054, *η*_p_^2^ = 0.05. For all conditions, children scored above chance level on the comprehension task on both the first and second post-test, all *p*s < 0.001, range *d*s = 1.49–2.83.

**TABLE 3:** Means (standard deviation) on all the tasks in the pre-test and post-tests for the four conditions.

		Iconic	No iconic	Tablet-only	Control
Pre-test	L1 vocabulary	108.13 (12.54)	108.67 (11.83)	105.77 (11.92)	108.88 (13.96)
	Phonological memory	10.08 (2.97)	11.33 (2.86)	11.08 (2.13)	10.16 (3.22)
	Selective attention	6.48 (0.65)	6.82 (0.58)	6.67 (0.64)	6.61 (0.82)
	Translation En-Du	3.41 (3.05)	3.59 (3.14)	3.98 (2.74)	2.81 (2.83)
First post-test	Translation En-Du	7.54 (5.14)	7.83 (4.94)	7.91 (4.63)	3.81 (3.21)
	Translation Du-En	6.09 (4.15)	6.54 (4.28)	6.64 (4.01)	3.16 (2.27)
	Comprehension	29.39 (5.78)	29.50 (6.13)	29.53 (6.40)	25.03 (6.66)
Second post-test	Translation En-Du	8.20 (4.98)	8.02 (4.92)	8.57 (4.61)	4.34 (3.22)
	Translation Du-En	6.57 (4.60)	6.44 (4.59)	6.75 (4.22)	3.47 (2.13)
	Comprehension	30.54 (6.26)	29.69 (6.61)	30.30 (6.55)	26.00 (6.04)

*Note.* The L1-vocabulary test is age-normed, with a mean of 100 and a standard deviation of 15. The maximum scores were 16 for the phonological-memory test, eight for the selective-attention test, 34 for each translation task, and 54 for the comprehension task (chance level for the latter task was 18).

### Effects of (Language) Learning Skills

**FIGURE 3:**
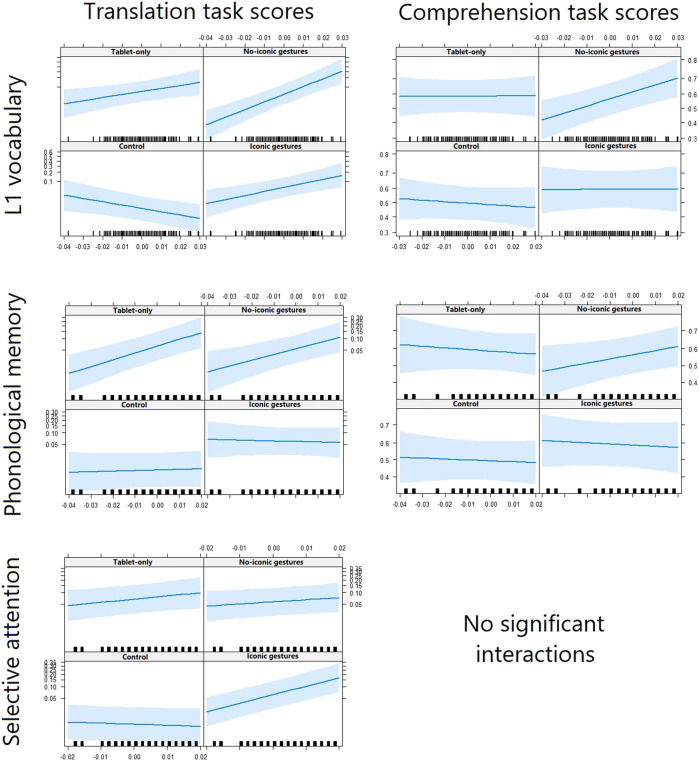
Relations between children’s English word-knowledge scores (*y*-axis) and (language) learning scores (*x*-axis), separated by condition and word-knowledge task.

First, we discuss the moderator effects of L1 vocabulary, phonological memory, and selective attention on the comparison of the experimental conditions versus the control condition. Then, we will discuss the moderator effects on the comparisons of the robot-assisted versus tablet-only conditions, and on the iconic-gestures versus no-iconic-gestures conditions. All outcomes can be found in the Appendix and the interactions are displayed in Figure 3.

#### Experimental Conditions vs. Control Condition

First, we investigated whether children’s (language) learning skills moderated the effect of the vocabulary intervention itself. We expected an effect of children’s (language) learning skills in the experimental conditions but not in the control condition, as only children in the experimental conditions received an L2 vocabulary training in which they could benefit from these skills. The models of the translation tasks showed statistically significant interactions between the moderator variables and condition. There were positive main effects for L1 vocabulary, *ß* = 72.47, *SE* = 8.99, *z* = 8.06, *p* < 0.001, phonological memory, *ß* = 22.13, *SE* = 8.07, *z* = 2.74, *p* = 0.006, and selective attention, *ß* = 36.46, *SE* = 6.47, *z* = 5.63, *p* < 0.001, but only for children in the experimental conditions, and not for those in the control condition, as expected. Note that the effects were only found for the translation tasks and not for the comprehension task.

#### Robot-Assisted Conditions vs. Tablet-Only Condition

Next, we investigated whether children’s (language) learning skills moderated the effect of the robot’s presence. We expected that the robot’s presence would particularly benefit children with poorer skills. Differences in translation task scores were found between the robot-assisted and tablet-only conditions for two of the three moderators, that is, L1 vocabulary, *ß* = −24.52, *SE* = 10.94, *z* = −2.24, *p* = 0.025, and phonological memory, *ß* = 26.72, *SE* = 10.53, *z* = 2.54, *p* = 0.011. Children with smaller L1 vocabularies knew more words in the tablet-only condition than in the robot-assisted conditions, while children with larger L1 vocabularies knew slightly more words in the robot-assisted conditions than in the tablet-only condition. The effect was opposite for phonological memory: Children with better phonological memory knew more words in the tablet-only condition than in the robot-assisted conditions, while children with poorer phonological memory knew more words in the robot-assisted conditions than in the tablet-only condition. This effect was in line with our expectation. No effects were found for the comprehension task or for selective attention, contrary to our expectation.

#### Iconic-Gestures Condition vs. No-Iconic-Gestures Condition

Last, we investigated whether children’s (language) learning skills moderated the effect of the robot’s gestures. We expected that the iconic gestures would benefit children with poorer skills most. Children with larger L1 vocabularies knew more target words in the no-iconic gestures condition, while children with smaller L1 vocabularies knew more words in the iconic-gestures condition. This was indicated by the models run on the translation tasks, *ß* = 31.99, *SE* = 9.16, *z* = 3.49, *p* < 0.001, and comprehension task, *ß* = 19.56, *SE* = 6.43, *z* = 3.05, *p* = 0.002. Similarly, children with better phonological memory knew more target words in the no-iconic-gestures condition, while children with poorer phonological memory knew more words in the iconic-gestures condition, but only for the translation tasks, *ß* = 40.52, *SE* = 14.99, *z* = 2.70, *p* = 0.007. Those effects were in line with our expectations. Selective attention showed an opposite pattern: Children with better selective attention showed higher performance in the condition in which the robot used iconic gestures than in the condition in which it did not, again only on the translation tasks, *ß* = −41.25, *SE* = 8.75, *z* = −4.71, *p* < 0.001.

## Discussion

The aim of our experiment was to investigate whether individual differences in L1 word knowledge, phonological memory, and selective attention moderated whether children benefit from the robot’s presence or its iconic gestures during robot-assisted L2 learning. Children in the present study were taught L2 English vocabulary through seven lessons in the form of tablet games, which they played either: 1) by themselves (the tablet-only condition); 2) together with a robot that used deictic gestures (the no-iconic-gestures condition); or 3) together with a robot that used both deictic and iconic gestures (the iconic-gestures condition). Furthermore, the children in the experimental conditions were compared to 4) a control group of children who did not play language games but played dancing games with the robot instead. Several statistically significant moderator effects were found, both expected and unexpected. We first discuss the general moderator effects of the (language) learning skills in the experimental conditions, then the moderator effects in the two robot-assisted conditions vs. the tablet-only condition, and lastly, the moderator effects in the iconic-gestures vs. no-iconic-gestures conditions. For the discussion of the general research question on the added value of the robot and its iconic gestures, see Vogt et al. (2019).

Regarding the overall effectiveness of the experimental conditions involving word-learning lessons compared to the control condition without word learning, we found the expected moderator effects: Children scoring high on L1 language knowledge, phonological memory, or selective attention, as assessed prior to the experiment, knew more words after the vocabulary lessons than children scoring low on these skills, in line with a vast body of literature that showed similar advantages in (second) language learning in general (Gathercole and Baddeley, 1990; Schmidt, 1990; Service, 1992; Robinson, 1995; Baddeley et al., 1998; Masoura and Gathercole, 2005; Gathercole, 2006; Wolter, 2006; Verhagen et al., 2019). No moderator effects were found in the control condition, which was expected because the control condition did not involve a word-learning intervention. The control condition, however, did involve an immediate and delayed post-test, similar to the experimental conditions. The lack of moderator effects in the control condition, therefore, supports the interpretation of the moderator effects in the experimental conditions as pertaining to the learning process, not to the test taking.

Regarding possible moderator effects between the three experimental conditions (i.e., the two robot-assisted conditions vs. the tablet-only condition), we expected the robot conditions to offer children a more naturalistic and supportive language-learning setting than the tablet-only condition (by grounding the interaction in the physical environment and allowing the learner to interact with another being; Barsalou, 2008; Ellis, 1999; Gallaway and Richard, 1994; Hockema and Smith, 2009; Iverson, 2010; Wellsby and Pexman, 2014). We expected that this would particularly benefit children poorer at language learning (i.e., children with smaller L1 vocabulary knowledge, smaller phonological memory capacity, and a lower level of selective attention). The robot’s presence particularly benefited children with larger L1 vocabularies or poorer phonological memory, while children with smaller L1 vocabularies or better phonological memory performed better in the tablet-only condition. These effects were only found for the translation tasks, and no effect was found for the comprehension tasks or for selective attention.

A possible explanation of why few effects were found is the prominent role of the tablet in our setup. It should be noted that the tablet was an essential device in the robot conditions, as technical limitations, in particular the lack of accurate speech perception (Kennedy et al., 2017) and object recognition for the type of robot we used (Wallbridge et al., 2017) required this extra device to enable interaction and communication. This may have limited the added value of the robot’s social presence. We were aware that using a tablet was a risk that could limit the benefits of the robot. We could have chosen to teleoperate our robot using WoZ, allowing us to make a highly responsive, adaptive robot. However, in view of the educational relevance of the current study, we wanted to design a robot that could function nearly autonomously, such that it was more representative of the type of robots that can currently be implemented in schools. Many technological developments are still needed before a robot’s full potential as an autonomous tutor in educational situations can be realized: Robots would need to be able to monitor the learner’s speech, knowledge, mental state, emotions, and movements, and adapt their own behavior accordingly. In the meantime, a balance needs to be found between making robots as effective as possible, without losing their autonomy. Perhaps we need to change the design process. Rather than first focusing on what tasks would be ideal from an instruction perspective, we should consider earlier in the process what qualities the robot does and does not have, and design tasks that match these qualities optimally.

With respect to the two robot-assisted conditions (iconic gestures vs. no iconic gestures), we expected the iconic gestures to further add to the naturalistic language learning environment and its visual support, and therefore, to particularly benefit children poorer at language learning. Children with smaller L1 vocabularies or poorer phonological memory capacity as assessed prior to the experiment knew more English words in the iconic-gestures condition compared to the no-iconic-gestures condition, while children with larger L1 vocabularies or larger phonological memory capacity knew more English words in the no-iconic-gestures condition compared to the iconic-gestures condition. Note that these moderator effects were observed in addition to positive main effects of both conditions compared to the control condition, and suggest that iconic gesturing in RALL may support children with weaker (language) learning abilities. Thus, the iconic gestures particularly benefited children with poorer (language) learning abilities as expected. However, they disadvantaged children with stronger (language) learning abilities. Perhaps, the iconic gestures distracted these children, who did not need these gestures to learn from the learning task (similar to Kennedy et al., 2015). Anecdotal evidence supports this suggestion, as experimenters occasionally observed that children looked away when the robot was making its gestures. We are currently systematically investigating this by looking into children’s engagement during the lessons (regarding both the learning task itself and the robot’s involvement) and by conducting additional analyses to identify subgroups of children who possibly benefited from the iconic gestures (e.g., depending on their age).

Selective attention showed an opposite pattern. Children high in selective attention knew more English words than children low in selective attention in the iconic-gestures condition compared to the no-iconic-gestures condition. Note again that the moderator effect was found in addition to positive main effects of both experimental conditions relative to the control condition. A possible explanation points again to the distracting effect the iconic gestures may have had on children’s word learning in this study (cf. Schmidt, 1990; Robinson, 1995). Children high in selective attention may have been better able to profit from the additional cues, which assumingly required attentional effort to perceive and interpret, and/or may have been less distracted by the extra information provided. Children low in selective attention may have been less capable in figuring out what the meaning was of the gestures and/or were more easily distracted by the gestures and the extra time it took the robot to perform these gestures. If true, this suggests that implementing iconic gestures benefits children with good attention skills, but disadvantages children with poorer attention skills. The results of benefiting some children while disadvantaging others highlights the importance of making adaptive robot-assisted lessons, which is in line with the conclusions of other recent studies that found limited benefits of a robot’s gestures for language learning (de Wit et al., 2020; Demir-Lira et al., 2020).

The present study reveals moderator effects of children’s (language) learning skills on the effectiveness of RALL. The findings, however, show a mixed pattern for the three (language) learning skills examined in this study. An open question is how children would respond to a robot (with or without iconic gestures) compared to a tablet or other non-robot condition if they have a mix of skills that are associated with opposite effects of robot-assisted instruction. For example, if a child both has a small L1 vocabulary and poor selective attention, it is unclear how they would respond to instruction by a robot with iconic gestures. On the one hand, the child could benefit from the iconic gestures. On the other hand, they would struggle to benefit from this additional, potentially distracting information. For future RALL research it is recommendable to identify profiles of skills in children and examine which profiles match best particular approaches to RALL. The overall results of the present study and Vogt et al. (2019) reveal that using robot tutoring in L2 learning programs for young children still has a long way to go. Designing the lesson series around the NAO robot, given the current state of technology, put severe constraints on the design of the lessons, required the use of a tablet for communication, and necessitated strong standardization. Traditional vocabulary training interventions may include more diverse activities that benefit learning and motivation, such as moving around and joint playing with objects. The robot was not yet capable of such activities in our study, and therefore, the only difference between the three experimental conditions was that children did not receive non-verbal support in the tablet-only condition through the robot’s social presence and its (iconic and) deictic gestures. Overall, children did not benefit from the robot’s presence, as was first reported in Vogt et al. (2019). The present study, however, reveals that children’s (language) learning abilities may moderate the effects of the presence of a robot and its iconic gestures. Future studies on RALL will likely benefit from technological advancements that allow RALL to incorporate more elements of effective traditional vocabulary training interventions, and to adapt optimally to individual learners’ skill profiles.

Our study is one of the first to investigate whether individual differences in children’s (language) learning abilities moderate the added value of a robot and its iconic gestures for L2 vocabulary learning in a multiple sessions and well-powered experiment. Taken together, the results suggest that the study of individual differences and moderators is highly relevant, as they showed that children’s (language) learning skills moderated the effect of the robot’s presence and iconic gestures: Depending on their (language) learning skills, some children benefited from the robot’s presence and iconic gestures, while some children appeared to be distracted by them. It is likely that the effects of the robot are different for different children and adaptation to children’s learning profiles is warranted. Indeed, one of the real advantages of robots is that they can play different roles for different types of learners if programmed to do so. The present results should be replicated before any firm conclusions can be drawn. The study of individual differences is standard practice in educational sciences and developmental psychology, and could add to studies on the design of adaptive robots for educational practice.

## Data Availability

The dataset and Benjamini-Hochberg calculations that support the findings of this study are openly available at OSF (https://doi.org/10.17605/OSF.IO/GSNEK). Further inquiries can be directed to the corresponding author.

## Footnotes

1
https://aspredicted.org/6k93k.pdf

2 One child from the Vogt et al. (2019) data was excluded from the analyses in the current paper, as this child had a different home language in addition to Dutch, which may interfere with the (language) learning variables studied here.

3
https://www.softbankrobotics.com/emea/en/nao

4 Video recordings of the gestures made by the robot can be found at https://tiu.nu/l2tor-gestures.

5 We also ran our model without moderator variables, and confirmed the results from Vogt et al. (2019; see Supplementary Table S1 for the results).

6 https://doi.org/10.17605/OSF.IO/GSNEK.
