# Supplementary material for: Individual Differences in Children’s (Language) Learning Skills Moderate Effects of Robot-Assisted Second Language Learning
Source: Front Robot AI. 2021 Aug 24;8:676248. doi: 10.3389/frobt.2021.676248 (PMC8421643; doi:10.3389/frobt.2021.676248)
Supplement: Supplementary file 1 [file DataSheet1.doc]

# Supplementary materials

Below, the tables for each of the models and each of the (language) learning skills (i.e., L1 vocabulary, phonological memory, and selective attention) can be found. The “ß” is an indicator of the effect size.

Table A.1

*Model without Moderator Variables: Results from the Mixed-Effects Logistic Regression Model with Accuracy Scores from the Translation Tasks or the Comprehension Task as the Dependent Variable, Condition as Between-Subjects Fixed Effect, and Time (and Target Language for Model Including the Translation Tasks) as Within-Subjects Fixed Effect*

|  | Translation tasks | | | | Comprehension task | | | |
| --- | --- | --- | --- | --- | --- | --- | --- | --- |
|  | *ß* | *SE* | *z* | *p* | *ß* | *SE* | *z* | *p* |
| (Intercept) | -3.19 | 0.43 | -7.46 | < .001 | 0.25 | 0.22 | 1.10 | .270 |
| Experimental vs. control | 1.90 | 0.38 | 4.97 | < .001 | 0.35 | 0.12 | 3.07 | .002 |
| Robot-assisted vs. tablet-only | 0.12 | 0.29 | 0.41 | .681 | 0.02 | 0.11 | 0.14 | .890 |
| Iconic vs. no-iconic gestures | -0.05 | 0.33 | -0.15 | .879 | -0.10 | 0.17 | -0.55 | .580 |
| Post-test | 0.16 | 0.05 | 3.28 | .001 | 0.05 | 0.04 | 1.09 | .276 |
| Language | -0.56 | 0.05 | -11.11 | < .001 | NA | NA | NA | NA |
| (Experimental vs. control) × time | -0.15 | 0.15 | -1.03 | .301 | -0.06 | 0.09 | -0.60 | .552 |
| (Robot-assisted vs. tablet-only) × time | 0.05 | 0.10 | 0.47 | .639 | 0.04 | 0.09 | 0.44 | .660 |
| (Iconic vs. no-iconic gestures) × time | -0.20 | 0.12 | -1.70 | .089 | -0.01 | 0.14 | -0.06 | .954 |

## Results for L1 Vocabulary

Table A.2

*Moderator Effects of L1 Vocabulary: Results from the Mixed-Effects Logistic Regression Model with Accuracy Scores from the English-Dutch Translation Task and Dutch-English Translation Task as Dependent Variables, Condition x× L1 Vocabulary as Between-Subjects Fixed Effects, and Time and Language as Within-Subjects Fixed Effects*

|  | *ß* | *SE* | *z* | *p* |
| --- | --- | --- | --- | --- |
| (Intercept) | -3.19 | 0.43 | -7.47 | < .001 |
| Experimental vs. control | 1.86 | 0.37 | 4.99 | < .001 |
| Robot-assisted vs. tablet-only | 0.20 | 0.28 | 0.70 | .483 |
| Iconic vs. no-iconic gestures | -0.10 | 0.32 | -0.31 | .757 |
| Time | 0.16 | 0.05 | 3.28 | .001 |
| Language | -0.56 | 0.05 | -11.11 | < .001 |
| L1 vocabulary | 25.19 | 8.96 | 2.81 | .005 |
| (Experimental vs. control) × time | -0.15 | 0.15 | -1.03 | .302 |
| (Robot-assisted vs. tablet-only) × time | 0.05 | 0.10 | 0.47 | .640 |
| (Iconic vs. no-iconic gestures) × time | -0.20 | 0.12 | -1.70 | .090 |
| (Experimental vs. control) × L1 vocabulary | 72.47 | 8.99 | 8.06 | < .001 |
| (Robot-assisted vs. tablet-only) × L1 vocabulary | -24.52 | 10.94 | -2.24 | .025 |
| (Iconic vs. no-iconic gestures) × L1 vocabulary | 31.99 | 9.16 | 3.49 | < .001 |

Table A.3

*Moderator Effects of L1 Vocabulary: Results from the Mixed-Effects Logistic Regression Model with Accuracy Scores from the Comprehension Task as a Dependent Variable, Condition × L1 Vocabulary as Between-Subjects Fixed Effects, and Time as a Within-Subjects Fixed Effect*

|  | *ß* | *SE* | *z* | *p* |
| --- | --- | --- | --- | --- |
| (Intercept) | 0.24 | 0.22 | 1.09 | .275 |
| Experimental vs. control | 0.34 | 0.11 | 3.05 | .002 |
| Robot-assisted vs. tablet-only | 0.02 | 0.11 | 0.22 | .829 |
| Iconic vs. no-iconic gestures | -0.11 | 0.17 | -0.66 | .513 |
| Time | 0.05 | 0.04 | 1.08 | .278 |
| L1 vocabulary | 4.01 | 4.13 | 0.97 | .332 |
| (Experimental vs. control) × time | -0.06 | 0.09 | -0.60 | .550 |
| (Robot-assisted vs. tablet-only) × time | 0.04 | 0.09 | 0.44 | .657 |
| (Iconic vs. no-iconic gestures) × time | -0.01 | 0.14 | -0.05 | .958 |
| (Experimental vs. control) × L1 vocabulary | 11.01 | 6.59 | 1.67 | .095 |
| (Robot-assisted vs. tablet-only) × L1 vocabulary | -9.86 | 6.67 | -1.48 | .140 |
| (Iconic vs. no-iconic gestures) × L1 vocabulary | 19.56 | 6.43 | 3.05 | .002 |

## Results for Phonological Memory

Table A.4

*Moderator Effects of Phonological Memory: Results from the Mixed-Effects Logistic Regression Model with Accuracy Scores from the English-Dutch Translation Task and Dutch-English Translation Task as Dependent Variables, Condition × Phonological Memory as Between-Subjects Fixed Effects, and Time and Language as Within-Subjects Fixed Effects*

|  | *ß* | *SE* | *z* | *p* |
| --- | --- | --- | --- | --- |
| (Intercept) | -3.23 | 0.43 | -7.57 | < .001 |
| Experimental vs. control | 1.82 | 0.38 | 4.80 | < .001 |
| Robot-assisted vs. tablet-only | 0.12 | 0.29 | 0.40 | .688 |
| Iconic vs. no-iconic gestures | -0.10 | 0.33 | -0.30 | .762 |
| Time | 0.16 | 0.05 | 3.18 | .001 |
| Language | -0.55 | 0.05 | -10.93 | < .001 |
| Phonological memory | 20.34 | 7.04 | 2.89 | .004 |
| (Experimental vs. control) × time | -0.16 | 0.15 | -1.08 | .282 |
| (Robot-assisted vs. tablet-only) × time | 0.06 | 0.10 | -0.56 | .575 |
| (Iconic vs. no-iconic gestures) × time | -0.18 | 0.12 | -1.52 | .129 |
| (Experimental vs. control) × phonological memory | 22.13 | 8.07 | 2.74 | .006 |
| (Robot-assisted vs. tablet-only) × phonological memory | 26.72 | 10.53 | 2.54 | .011 |
| (Iconic vs. no-iconic gestures) × phonological memory | 40.52 | 14.99 | 2.70 | .007 |

Table A.5

*Moderator Effects of Phonological Memory: Results from the Mixed-Effects Logistic Regression Model with Accuracy Scores from the Comprehension Task as a Dependent Variable, Condition × Phonological Memory as Between-Subjects Fixed Effects, and Time as a Within-Subjects Fixed Effect*

|  | *ß* | *SE* | *z* | *p* |
| --- | --- | --- | --- | --- |
| (Intercept) | 0.24 | 0.23 | 1.05 | .294 |
| Experimental vs. control | 0.34 | 0.12 | 2.97 | .003 |
| Robot-assisted vs. tablet-only | 0.04 | 0.12 | 0.38 | .708 |
| Iconic vs. no-iconic gestures | -0.09 | 0.18 | -0.49 | .621 |
| Time | 0.05 | 0.04 | 1.09 | .277 |
| Phonological memory | 0.32 | 4.13 | 0.08 | .939 |
| (Experimental vs. control) × time | -0.06 | 0.09 | -0.60 | .552 |
| (Robot-assisted vs. tablet-only) × time | 0.04 | 0.09 | 0.44 | .659 |
| (Iconic vs. no-iconic gestures) × time | -0.01 | 0.14 | -0.06 | .956 |
| (Experimental vs. control) × phonological memory | 3.21 | 5.89 | 0.55 | .586 |
| (Robot-assisted vs. tablet-only) × phonological memory | -7.45 | 6.12 | -1.22 | .224 |
| (Iconic vs. no-iconic gestures) × phonological memory | 12.85 | 5.67 | 2.27 | .023 |

## Results for Selective Attention

Table A.6

*Moderator Effects of Selective Attention: Results from the Mixed-Effects Logistic Regression Model with Accuracy Scores from the English-Dutch Translation Task and Dutch-English Translation Task as Dependent Variables, Condition × Selective Attention as Between-Subjects Fixed Effects, and Time and Language as Within-Subjects Fixed Effects*

|  | *ß* | *SE* | *z* | *p* |
| --- | --- | --- | --- | --- |
| (Intercept) | -3.19 | 0.43 | -7.47 | < .001 |
| Experimental vs. control | 1.90 | 0.38 | 5.04 | < .001 |
| Robot-assisted vs. tablet-only | 0.13 | 0.29 | 0.46 | .649 |
| Iconic vs. no-iconic gestures | -0.09 | 0.33 | -0.26 | .793 |
| Time | 0.16 | 0.05 | 3.28 | .001 |
| Language | -0.56 | 0.05 | -11.11 | < .001 |
| Selective attention | 20.58 | 6.66 | 3.09 | .002 |
| (Experimental vs. control) × time | -0.15 | 0.15 | -1.03 | .302 |
| (Robot-assisted vs. tablet-only) × time | 0.05 | 0.10 | 0.47 | .640 |
| (Iconic vs. no-iconic gestures) × time | -0.20 | 0.12 | -1.70 | .089 |
| (Experimental vs. control) × selective attention | 36.46 | 6.47 | 5.63 | < .001 |
| (Robot-assisted vs. tablet-only) × selective attention | -13.53 | 10.42 | -1.30 | .194 |
| (Iconic vs. no-iconic gestures) × selective attention | -41.25 | 8.75 | -4.71 | < .001 |

Table A.7

*Moderator Effects of Selective Attention: Results from the Mixed-Effects Logistic Regression Model with Accuracy Scores from the Comprehension Task as a Dependent Variable, Condition × Selective Attention as Between-Subjects Fixed Effects, and Time as a Within-Subjects Fixed Effect*

|  | *ß* | *SE* | *z* | *p* |
| --- | --- | --- | --- | --- |
| (Intercept) | 0.28 | 0.23 | 1.26 | .210 |
| Experimental vs. control | 0.30 | 0.13 | 2.30 | .021 |
| Robot-assisted vs. tablet-only | 0.02 | 0.13 | 0.17 | .862 |
| Iconic vs. no-iconic gestures | -0.21 | 0.19 | -1.10 | .272 |
| Time | 0.03 | 0.05 | 0.59 | .557 |
| Selective attention | 9.06 | 4.61 | 1.96 | .050 |
| (Experimental vs. control) × time | -0.01 | 0.12 | -0.07 | .946 |
| (Robot-assisted vs. tablet-only) × time | 0.05 | 0.10 | 0.47 | .638 |
| (Iconic vs. no-iconic gestures) × time | -0.02 | 0.15 | -0.12 | .908 |
| (Experimental vs. control) × selective attention | 10.40 | 8.07 | 1.29 | .197 |
| (Robot-assisted vs. tablet-only) × selective attention | 11.11 | 8.03 | 1.38 | .166 |
| (Iconic vs. no-iconic gestures) × selective attention | -7.63 | 8.09 | -0.94 | .346 |

## Benjamini-Hochberg Procedure

Table A.8

*Benjamini-Hochberg Procedure for Moderator Effects of L1 Vocabulary on the Translation Tasks (Full Model in Table A.2)*

| Test | *p*-value | rank | critical value |
| --- | --- | --- | --- |
| (Intercept) | < .001* | 1 | .003846154 |
| Experimental vs. control | < .001* | 2 | .007692308 |
| Language | < .001* | 3 | .011538462 |
| (Experimental vs. control) × L1 vocabulary | < .001* | 4 | .015384615 |
| (Iconic vs. no-iconic gestures) × L1 vocabulary | < .001* | 5 | .019230769 |
| Time | .001* | 6 | .023076923 |
| L1 vocabulary | .005* | 7 | .026923077 |
| (Robot-assisted vs. tablet-only) × L1 vocabulary | .025* | 8 | .030769231 |
| (Iconic vs. no-iconic gestures) × time | .09 | 9 | .034615385 |
| (Experimental vs. control) × time | .302 | 10 | .038461538 |
| Robot-assisted vs. tablet-only | .483 | 11 | .042307692 |
| (Robot-assisted vs. tablet-only) × time | .64 | 12 | .046153846 |
| Iconic vs. no-iconic gestures | .757 | 13 | .05 |

*Note.* Effects considered significant (the test with the largest *p*-value that is smaller than the critical value) are marked with an asterisk.

Table A.9

*Benjamini-Hochberg Procedure for Moderator Effects of L1 Vocabulary on the Comprehension Task (Full Model in Table A.3)*

| Test | *p*-value | rank | critical value |
| --- | --- | --- | --- |
| Experimental vs. control | .002* | 1 | .004166667 |
| (Iconic vs. no-iconic gestures) × L1 vocabulary | .002* | 2 | .008333333 |
| (Experimental vs. control) × L1 vocabulary | .095 | 3 | .0125 |
| (Robot-assisted vs. tablet-only) × L1 vocabulary | .14 | 4 | .016666667 |
| (Intercept) | .275 | 5 | .020833333 |
| Time | .278 | 6 | .025 |
| L1 vocabulary | .332 | 7 | .029166667 |
| Iconic vs. no-iconic gestures | .513 | 8 | .033333333 |
| (Experimental vs. control) × time | .55 | 9 | .0375 |
| (Robot-assisted vs. tablet-only) × time | .657 | 10 | .041666667 |
| Robot-assisted vs. tablet-only | .829 | 11 | .045833333 |
| (Iconic vs. no-iconic gestures) × time | .958 | 12 | .05 |

*Note.* Effects considered significant (the test with the largest *p*-value that is smaller than the critical value) are marked with an asterisk.

Table A.10

*Benjamini-Hochberg Procedure for Moderator Effects of Phonological Memory on the Translation Tasks (Full Model in Table A.4)*

| Test | *p*-value | rank | critical value |
| --- | --- | --- | --- |
| (Intercept) | < .001* | 1 | .003846154 |
| Experimental vs. control | < .001* | 2 | .007692308 |
| Language | < .001* | 3 | .011538462 |
| Time | .001* | 4 | .015384615 |
| Phonological memory | .004* | 5 | .019230769 |
| (Experimental vs. control) × phonological memory | .006* | 6 | .023076923 |
| (Iconic vs. no-iconic gestures) × phonological memory | .007* | 7 | .026923077 |
| (Robot-assisted vs. tablet-only) × phonological memory | .011* | 8 | .030769231 |
| (Iconic vs. no-iconic gestures) × time | .129 | 9 | .034615385 |
| (Experimental vs. control) × time | .282 | 10 | .038461538 |
| (Robot-assisted vs. tablet-only) × time | .575 | 11 | .042307692 |
| Robot-assisted vs. tablet-only | .688 | 12 | .046153846 |
| Iconic vs. no-iconic gestures | .762 | 13 | .05 |

*Note.* Effects considered significant (the test with the largest *p*-value that is smaller than the critical value) are marked with an asterisk.

Table A.11

*Benjamini-Hochberg Procedure for Moderator Effects of Phonological Memory on the Comprehension Task (Full Model in Table A.5)*

| Test | *p*-value | rank | critical value |
| --- | --- | --- | --- |
| Experimental vs. control | .003* | 1 | .004166667 |
| (Iconic vs. no-iconic gestures) × phonological memory | .023 | 2 | .008333333 |
| (Robot-assisted vs. tablet-only) × phonological memory | .224 | 3 | .0125 |
| Time | .277 | 4 | .016666667 |
| (Intercept) | .294 | 5 | .020833333 |
| (Experimental vs. control) × time | .552 | 6 | .025 |
| (Experimental vs. control) × phonological memory | .586 | 7 | .029166667 |
| Iconic vs. no-iconic gestures | .621 | 8 | .033333333 |
| (Robot-assisted vs. tablet-only) × time | .659 | 9 | .0375 |
| Robot-assisted vs. tablet-only | .708 | 10 | .041666667 |
| Phonological memory | .939 | 11 | .045833333 |
| (Iconic vs. no-iconic gestures) × time | .956 | 12 | .05 |

*Note.* Effects considered significant (the test with the largest *p*-value that is smaller than the critical value) are marked with an asterisk.

Table A.12

*Benjamini-Hochberg Procedure for Moderator Effects of Selective Attention on the Translation Tasks (Full Model in Table A.6)*

| Test | *p*-value | rank | critical value |
| --- | --- | --- | --- |
| (Intercept) | < .001* | 1 | .003846154 |
| Experimental vs. control | < .001* | 2 | .007692308 |
| Language | < .001* | 3 | .011538462 |
| (Experimental vs. control) × selective attention | < .001* | 4 | .015384615 |
| (Iconic vs. no-iconic gestures) × selective attention | < .001* | 5 | .019230769 |
| Time | .001* | 6 | .023076923 |
| Selective attention | .002* | 7 | .026923077 |
| (Iconic vs. no-iconic gestures) × time | .089 | 8 | .030769231 |
| (Robot-assisted vs. tablet-only) × selective attention | .194 | 9 | .034615385 |
| (Experimental vs. control) × time | .302 | 10 | .038461538 |
| (Robot-assisted vs. tablet-only) × time | .64 | 11 | .042307692 |
| Robot-assisted vs. tablet-only | .649 | 12 | .046153846 |
| Iconic vs. no-iconic gestures | .793 | 13 | .05 |

*Note.* Effects considered significant (the test with the largest *p*-value that is smaller than the critical value) are marked with an asterisk.

Table A.13

*Benjamini-Hochberg Procedure for Moderator Effects of Selective Attention on the Comprehension Task (Full Model in Table A.7)*

| Test | *p*-value | rank | critical value |
| --- | --- | --- | --- |
| Experimental vs. control | .021 | 1 | .004166667 |
| Selective attention | .05 | 2 | .008333333 |
| (Robot-assisted vs. tablet-only) × selective attention | .166 | 3 | .0125 |
| (Experimental vs. control) × selective attention | .197 | 4 | .016666667 |
| (Intercept) | .21 | 5 | .020833333 |
| Iconic vs. no-iconic gestures | .272 | 6 | .025 |
| (Iconic vs. no-iconic gestures) × selective attention | .346 | 7 | .029166667 |
| Time | .557 | 8 | .033333333 |
| (Robot-assisted vs. tablet-only) × time | .638 | 9 | .0375 |
| Robot-assisted vs. tablet-only | .862 | 10 | .041666667 |
| (Iconic vs. no-iconic gestures) × time | .908 | 11 | .045833333 |
| (Experimental vs. control) × time | .946 | 12 | .05 |

*Note.* Effects considered significant (the test with the largest *p*-value that is smaller than the critical value) are marked with an asterisk.
